# FROM MICRO TO MACRO: PSYCHOSOCIAL AND BEHAVIORAL PREDICTORS OF COGNITIVE AGING ACROSS DAYS AND DECADES

**DOI:** 10.1093/geroni/igad104.0362

**Published:** 2023-12-21

**Authors:** Jeremy Hamm, Jacqueline Mogle

## Abstract

Healthier cognitive aging is multifactorial, shaped in part by modifiable psychosocial and behavioral predictors. Critically, these predictors exhibit day-to-day and year-to-year fluctuations that could be harnessed to promote better cognitive functioning with age. The present studies thus address short- and long-term differences in psychosocial (perceived control, relationship quality) and behavioral factors (light physical activity, muscle strength) as predictors of normative and non-normative cognitive aging. Cerino et al. use data from the National Study of Daily Experiences to demonstrate how daily perceptions of control over daily stressors relate to episodic memory and executive functioning across age. Jang and Mogle examine the association between daily appraisals of relationship quality and subjective cognitive decline as driven by daily stressful experiences. Hamm et al. use data from the Midlife in the United States Study to investigate how changes in light physical activity across 9-years predict changes in episodic memory and executive functioning beyond more vigorous forms of activity, particularly for older adults. Finally, McGrath et al. use 12-year data from the Health and Retirement Study to investigate a multidimensional measure of maximum voluntary muscle strength as an indicator of elevated cognitive impairment risk among older adults. This symposium thus integrates new research from deeply-phenotyped samples on psychosocial and behavioral factors implicated in cognitive aging and contributes to a more nuanced understanding of how healthier cognitive functioning across short (days) and long (decades) time spans is shaped by beliefs, social dynamics, and behaviors that are amenable to change across the adult lifespan.

